# Genetic background (DDD/Sgn versus C57BL/6J) strongly influences postnatal growth of male mice carrying the *A*^*y*^ allele at the agouti locus: identification of quantitative trait loci associated with diabetes and body weight loss

**DOI:** 10.1186/1471-2156-14-35

**Published:** 2013-05-04

**Authors:** Jun-ichi Suto, Kunio Satou

**Affiliations:** 1 Agrogenomics Research Center, National Institute of Agrobiological Sciences, Tsukuba, Ibaraki 305-8634, Japan; 2 Viral Disease and Epidemiology Research Division, National Institute of Animal Health, Tsukuba, Ibaraki 305-0856, Japan

**Keywords:** *A*^*y*^ allele, Body weight loss, DDD mice, Diabetes mellitus, Growth analysis, Quantitative trait locus (QTL)

## Abstract

**Background:**

Mice carrying the *A*^*y*^ allele at the agouti locus become obese and are heavier than their non-*A*^*y*^ littermates. However, this does not hold true for the genetic background of the DDD mouse strain. At 22 weeks of age, DDD.Cg-*A*^*y*^ females are heavier than DDD females, whereas DDD.Cg-*A*^*y*^ males are lighter than DDD males. This study aimed to determine the possible cause and identify the genes responsible for the lower body weight of DDD.Cg-*A*^*y*^ males.

**Results:**

Growth curves of DDD.Cg-*A*^*y*^ mice were analyzed and compared with those of B6.Cg-*A*^*y*^ mice from 5 to 25 weeks. In DDD.Cg-*A*^*y*^ males, body weight gain stopped between 16 and 17 weeks and the body weight gradually decreased; thus, the lower body weight was a consequence of body weight loss. Quantitative trait locus (QTL) mapping was performed in backcrossed (BC) males of DDD × (B6 × DDD.Cg-*A*^*y*^) F_1_-*A*^*y*^ mice. For the body weight at 25 weeks, significant QTLs were identified on chromosomes 1 and 4. The DDD allele was associated with a lower body weight at both loci. In particular, the QTL on chromosome 4 interacted with the *A*^*y*^ allele. Furthermore, suggestive QTLs for plasma glucose and high molecular weight adiponectin levels were coincidentally mapped to chromosome 4. The DDD allele was associated with increased glucose and decreased adiponectin levels. When the body weight at 25 weeks and plasma glucose levels were considered as dependent and independent variables, respectively, BC *A*^*y*^ males were classified into two groups according to statistical analysis using the partition method. Mice of one group had significantly higher glucose and lower adiponectin levels than those of the other group and exhibited body weight loss as observed with DDD-*A*^*y*^ males.

**Conclusions:**

The lower body weight of DDD.Cg-*A*^*y*^ male mice was a consequence of body weight loss. Diabetes mellitus has been suggested to be a possible contributory factor causing body weight loss. The QTL on distal chromosome 4 contained the major responsible genes. This QTL interacted with the *A*^*y*^ allele, implying the reason why body weight loss occurs in DDD.Cg-*A*^*y*^ but not in DDD males.

## Background

Traditionally, five single gene obesity mutations, *Cpe*^*fat*^, *Tub*^*tub*^, *Lep*^*ob*^, *Lepr*^*db*^, and *A*^*y*^, have been identified in mice [1]. Among these, only the *A*^*y*^ allele is dominant and homozygous lethal; therefore, living *A*^*y*^ mice are invariably heterozygotes. Obesity in *A*^*y*^ mice is moderate and occurs late compared with that in the other four mutants. *A*^*y*^ mice also show metabolic abnormalities such as hyperglycemia and marked hyperinsulinemia, making them a suitable animal model system for studying obesity-associated diabetes mellitus in humans.

In normal mice, the agouti gene is expressed only in the skin [2,3], and it regulates pigmentation by acting as an inverse agonist of the melanocortin 1 receptor [4,5]. However, in *A*^*y*^ mice, the *A*^*y*^ allele is associated with a large deletion, which causes agouti gene expression to be aberrantly controlled by the unrelated *Raly* gene promoter and results in its ectopic overexpression [3,6-8]. Thus, *A*^*y*^ mice have a yellow coat color and develop maturity-onset obesity. Obesity in *A*^*y*^ mice is thought to be a consequence of the agouti protein acting as a constitutive antagonist of the melanocortin 3 receptors (MC3R) and melanocortin 4 receptor (MC4R) by mimicking the actions of the agouti-related protein [9-11].

Till date, two mouse strains congenic for the *A*^*y*^ allele are available: B6.Cg-*A*^*y*^ (C57BL/6J background; hereafter B6-*A*^*y*^) and KK.Cg-*A*^*y*^ (KK/Ta background; hereafter KK-*A*^*y*^). We developed a third mouse strain congenic for the *A*^*y*^ allele in an inbred DDD/Sgn (hereafter DDD) strain background, (i.e., DDD.Cg-*A*^*y*^; hereafter DDD-*A*^*y*^) [12]. DDD-*A*^*y*^ females are significantly obese compared with B6-*A*^*y*^ and KK-*A*^*y*^ females [13].

From our previous observations, DDD-*A*^*y*^ females (average body weight of 62.1 g at 22 weeks) are heavier than DDD females (37.6 g), whereas DDD-*A*^*y*^ males (36.0 g) are lighter than DDD males (40.4 g). Surprisingly, within a DDD background, the *A*^*y*^ allele increased the female body weight, whereas it reduced the male body weight. Within B6 and KK backgrounds, *A*^*y*^ males are heavier than their non-*A*^*y*^ male littermates.

To confirm this unusual phenomenon, we conducted additional detailed analyses of the body weight and growth in the DDD-*A*^*y*^ strain in comparison with those in B6-*A*^*y*^ and reciprocal F_1_ strains. We also conducted some pathophysiological analyses to determine possible causes of the lower body weight. In addition, we conducted quantitative trait locus (QTL) mapping analysis to identify genes associated with the lower body weight within the DDD background based on the assumption that the lower body weight was a heritable phenomenon.

## Methods

### Mouse strains

The inbred DDD mouse strain was maintained at the National Institute of Agrobiological Sciences (NIAS, Tsukuba, Ibaraki, Japan). The inbred B6 mouse strain was purchased from Clea Japan, Inc. (Tokyo, Japan), and the congenic B6-*A*^*y*^ mouse strain was purchased from the Jackson Laboratory (Bar Harbor, ME, USA). The congenic DDD-*A*^*y*^ mouse strain was established by introgression of the *A*^*y*^ allele from the B6-*A*^*y*^ strain into the DDD strain by backcrossing for 12 generations. Because the original DDD strain had an albino coat, congenic DDD-*A*^*y*^ mice were further intercrossed between yellow (*A*^*y*^) and agouti (*A*) littermates to eliminate the *Tyr*^*c*^ allele.

In this study, agouti littermates were used as DDD control mice. Hereafter, DDD-*A*^*y*^ and B6-*A*^*y*^ are collectively referred to as *A*^*y*^ mice. Likewise, their control littermates, DDD and B6, were designated as non-*A*^*y*^ mice. Mice homozygous for the *A*^*y*^ allele were embryonic lethal; thus, all *A*^*y*^ mice used in this study were heterozygotes. *A*^*y*^ mice were distinguishable from non-*A*^*y*^ mice because *A*^*y*^ mice had yellow coats (as opposed to non-*A*^*y*^ mice with agouti or black coats).

We also analyzed reciprocal F_1_ mice produced by a cross between DDD (−*A*^*y*^) and B6 (−*A*^*y*^) strains. (♀DDD × ♂B6-*A*^*y*^) F_1_ mice were designated DB, and (♀B6 × ♂DDD-*A*^*y*^) F_1_ were designated BD. Following the designations of inbred strains, DB-*A*^*y*^ and BD-*A*^*y*^ mice were referred to as *A*^*y*^ mice and their agouti littermates were referred to as non-*A*^*y*^ mice. The mouse strain designations used in this study are summarized in Table 1.

**Table 1:** Designations of mouse strains used for growth analyses

**Designation**	**Synonymous designation**	**Strain background**	**Coat color (agouti genotype)**	**Sex**	**No. of mice used**
DDDYM	DDD-*A*^*y*^ male	DDD	Yellow (*A*^*y*^)	Male	14
DDDYF	DDD-*A*^*y*^ female	DDD	Yellow (*A*^*y*^)	Female	23
DDDAM	DDD male	DDD	Agouti (*A*)	Male	17
DDDAF	DDD female	DDD	Agouti (*A*)	Female	14
B6YM	B6-*A*^*y*^ male	B6	Yellow (*A*^*y*^)	Male	11
B6YF	B6-*A*^*y*^ female	B6	Yellow (*A*^*y*^)	Female	17
B6AM	B6 male	B6	Black (*a*)	Male	11
B6AF	B6 female	B6	Black (*a*)	Female	15
DBYM	DB-*A*^*y*^ male	(♀DDD × ♂B6) F_1_	Yellow (*A*^*y*^)	Male	9
DBYF	DB-*A*^*y*^ female	(♀DDD × ♂B6) F_1_	Yellow (*A*^*y*^)	Female	10
DBAM	DB male	(♀DDD × ♂B6) F_1_	Agouti (*A*)	Male	9
DBAF	DB female	(♀DDD × ♂B6) F_1_	Agouti (*A*)	Female	10
BDYM	BD-*A*^*y*^ male	(♀B6 × ♂DDD) F_1_	Yellow (*A*^*y*^)	Male	12
BDYF	BD-*A*^*y*^ male	(♀B6 × ♂DDD) F_1_	Yellow (*A*^*y*^)	Female	16
BDAM	BD male	(♀B6 × ♂DDD) F_1_	Agouti (*A*)	Male	22
BDAF	BD female	(♀B6 × ♂DDD) F_1_	Agouti (*A*)	Female	15

Coat color of B6 males and B6 females is actually black but is classified as agouti (i.e., *A*) for convenience.

Mice were weaned at 4 weeks of age, and 4–5 mice were housed together during the experimental period. All the mice were maintained in a specific pathogen-free facility with a regular light cycle and controlled temperature and humidity. Food (CRF-1; Oriental yeast Co. Ltd., Tokyo, Japan) and water were freely available throughout the experimental period. All the animal experiments were conducted in accordance with the guidelines of the Institutional Animal Care and Use Committee of NIAS.

### Body weight measurements and growth analysis

Body weights were weekly measured from 5 to 25 weeks of age and were determined to the nearest 0.01 g using an electronic balance. The average body weights of mice from 5 to 25 weeks were modeled as the study outcomes using the following three equations:

(1) $$\mathrm{Body}\mathrm{weight}=a\times \left(1-e^{-b\times \mathrm{Week}}\right)+\epsilon$$

(2) $$\mathrm{Log}\left(\mathrm{Body}\mathrm{weight}\right)=a+b\times \mathrm{Log}\left(\mathrm{Week}\right)+\epsilon$$

(3) $$\begin{array}{l} \mathrm{Log}\left(\mathrm{Body}\mathrm{weight}\right)=a+b\times \mathrm{Log}\left(\mathrm{Week}\right)+c\\ \;\times {\left[\mathrm{Log}\left(\mathrm{Week}\right)\right]}^{2}+\epsilon \end{array}$$

Eqs. 1 and 2 are known as the von Bertalanffy growth model [14] and the elasticity model [15], respectively. In Eq. 1, the body weight at birth was assumed to be zero and the growth rate was considered to be proportional to the maximum size minus the current size. Thus, the body weight reached an upper limit value over time. In Eq. 2, elasticity was a coefficient estimated from a linear regression equation where both dependent and independent variables were presented as natural logs. In this model, the growth rate was proportional to the current ratio of the body weight to age. In Eq. 3, we added a quadratic term to Eq. 2 to fit the curves better to the observed data. In Eqs. 2 and 3, the body weight was expressed as *Exp* (*a*) at 1 week of age using the value of parameter *a*.

For these analyses, the independent variable of time was treated as a continuous variable and mouse characteristics (strain, agouti, sex, and their interactions) were treated as nominal variables.

For the agouti genotype (i.e., whether the mouse had the *A*^*y*^ allele), mice with or without the *A*^*y*^ allele were designated Y and A, respectively. With regard to sex, males and females were designated M and F, respectively. With regard to strains, mice were classified into one of the following four strains: DDD, B6, BD, and DB. Agouti, sex, and strain were used as independent variables and designated Strain*Agouti*Sex. All the mice were classified into one of the following 16 categories: DDDYM, DDDYF, DDDAM, DDDAF, B6YM, B6YF, B6AM, B6AF, DBYM, DBYF, DBAM, DBAF, BDYM, BDYF, BDAM, and BDAF.

In this study, growth models were designated models 1, 2, and 3, as described by Eqs. 1, 2, and 3, respectively. Each of the 16 Strain*Agouti*Sex categories was analyzed using the three models. In model 1, the maximum likelihood estimates for parameter values were obtained by minimizing the deviance in the observed data by weighting by the number of mice. The 95% confidence intervals (CIs) were determined using the profile likelihood. In models 2 and 3, the optimum estimates and 95% CI for the parameters were determined using the least-squares method weighted by the number of mice. Statistical significance was set at the α = 0.05 level. Data analysis used JMP ver. 9.0.1 statistical software (SAS Institute Inc., Cary, NC, USA).

### Blood phenotype analyses

At 25 weeks of age, mice were euthanized with an overdose of ether after they were fasted for 4 h. Whole blood was drawn from the heart into a plastic tube using heparin as an anticoagulant. Blood glucose (BGLC) levels were then determined using Glutest Pro R (Sanwa Kagaku Kenkyusho, Aichi, Japan) according to the manufacturer’s instructions. Sample tubes were centrifuged at 7,000 rpm for 5 min at 4°C to separate plasma. The plasma samples were maintained at −70°C until use. Plasma glucose (PGLC) levels were determined using a clinical colorimetric kit (Glucose C-II Test Wako, Wako Pure Chemical Industries, Osaka, Japan). Plasma total-cholesterol (CHO) and triglyceride (TG) levels were determined using Cholesterol E Test Wako and Triglyceride E Test Wako (Wako Pure Chemical Industries). Plasma high-molecular-weight adiponectin (HMW adiponectin) levels were determined using the Mouse/Rat High Molecular Weight Adiponectin ELISA Kit (AKMAN-011, Shibayagi, Gunma, Japan). For statistical comparisons between groups, the Tukey–Kramer honestly significant difference (HSD) test was used. P values <0.05 were considered statistically significant.

### Histology

The liver, pancreas, and adipose tissues were removed at necropsy from parental strains of both the sexes. Formalin-fixed tissues were embedded in paraffin, sectioned, and stained with hematoxylin and eosin.

### Other phenotyping

The anal–nasal length of each mouse was measured using a pair of digital calipers to the nearest 0.01 mm just after blood collection to avoid rigor mortis. Body length was defined as the anal–nasal length. Body mass index (BMI) was calculated as follows: body weight (g)/[body length (mm)]^2^ × 10^3^. BMI was used to estimate the degree of obesity.

### QTL mapping

QTL mapping analysis was conducted to identify genes possibly associated with the lower body weight of DDD-*A*^*y*^ males. We produced 196 backcross (BC) males, comprising 100 *A*^*y*^ (BC *A*^*y*^) and 96 non-*A*^*y*^ (BC non-*A*^*y*^) males, from a genetic cross of ♀DDD × ♂(♀B6 × ♂DDD-A^*y*^) F_1_-*A*^*y*^ mice. This backcrossing was chosen because neither BD-*A*^*y*^ males nor DB-*A*^*y*^ males showed body weight loss and the DDD alleles involved in this phenomenon were considered to be recessive to the B6 allele. QTL analysis was primarily conducted in 100 BC *A*^*y*^ males; 96 BC non-*A*^*y*^ males were genotyped for all markers on chromosomes 1 and 4.

Genomic DNA isolation and genotyping of microsatellite markers used the procedures described in our previous study [13]. Microsatellite markers used in this study are listed in Table 2 with their map positions (cM) calculated using 196 BC males. X chromosome-linked markers were not genotyped because they were not informative with this genetic cross.

**Table 2:** Genetic markers used in this study and their map positions

**Marker**	**Map position**^**a**^	**Marker**	**Map position**	**Marker**	**Map position**	**Marker**	**Map position**
Chromosome 1		Chromosome 6		Chromosome 11		Chromosome 16	
*D1Mit231*	0	*D6Mit116*	0	*D11Mit236*	0	*D16Mit131*	0
*D1Mit303*	24.9	*D6Mit224*	17.3	*D11Mit36*	45.9	*D16Mit57*	6.8
*D1Mit10*	41.0	*D6Mit188*	27.1	*D11Mit124*	51.4	*D16Mit136*	25.8
*D1Mit102*	55.5	*D6Mit39*	38.9	*D11Mit61*	65.6	*D16Mit139*	38.8
*D1Mit16*	67.5	*D6Mit108*	44.4			*D16Mit49*	44.4
*D1Mit291*	73.4	*D6Mit256*	45.3	Chromosome 12			
		*D6Mit259*	56.1	*D12Mit136*	0	Chromosome 17	
Chromosome 2				*D12Mit172*	6.9	*D17Mit164*	0
*D2Mit312*	0	Chromosome 7.1		*D12Mit156*	20.7	*D17Mit176*	9.4
*D2Mit296*	21.2	*D7Mit250*	0	*D12Mit259*	26.5	*D17Mit139*	13.5
*D2Mit92*	40.8			*D12Mit141*	47.3	*D17Mit93*	41.1
		Chromosome 7.2		*D12Nds2*	49.3	*D17Mit123*	60.3
Chromosome 3		*D7Mit362*	0				
*D3Mit203*	0			Chromosome 13		Chromosome 18	
*D3Mit25*	19.7	Chromosome 8		*D13Mit207*	0	*D18Mit21*	0
*D3Mit212*	26.5	*D8Mit191*	0	*D13Mit64*	16.1	*D18Mit149*	12.0
		*D8Mit205*	4.5	*D13Mit110*	46.1	*D18Mit152*	22.0
Chromosome 4		*D8Mit249*	13.6	*D13Mit213*	53.0	*D18Mit25*	71.1
*D4Mit1*	0	*D8Mit183*	28.4	*D13Mit171*	56.3		
*D4Mit178*	16.5					Chromosome 19	
*D4Mit166*	27.8	Chromosome 9		Chromosome 14		*D19Mit32*	0
*D4Mit12*	48.8	*D9Mit59*	0	*D14Mit64*	0	*D19Mit91*	34.3
*D4Mit234*	60.6	*D9Mit191*	13.5	*D14Mit193*	10.5	*D19Mit35*	35.1
*D4Mit232*	60.6	*D9Mit207*	19.9	*D14Mit165*	35.7		
*D4Mit42*	60.7	*D9Mit198*	42.2				
		*D9Mit212*	55.5	Chromosome 15			
Chromosome 5				*D15Mit174*	0		
*D5Mit267*	0	Chromosome 10		*D15Mit184*	14.2		
*D5Mit113*	16.6	*D10Mit188*	0	*D15Mit193*	79.7		
*D5Mit239*	23.0	*D10Mit183*	3.1				
*D5Mit161*	34.7	*D10Mit42*	29.0				
*D5Mit221*	52.6	*D10Mit95*	33.3				

^a^Map positions (cM) were based on a linkage map calculated using 196 BC males.

Notably, chromosome 7 is divided into two parts. Because of the introgression of the *Tyr* locus from the B6 strain, an intermediate part of the DDD genome on chromosome 7 is replaced by a B6 genome in DDD-*A*^*y*^ mice. In this study, a region proximal to the B6 region was defined as “chromosome 7.1 (*D7Mit250*)” whereas a region distal to the B6 region was defined as “chromosome 7.2 (*D7Mit362*).”

QTL analysis was conducted using R/qtl [16,17]. Threshold logarithm of odds (LOD) scores for suggestive (P < 0.63) and significant (P < 0.05) linkages were determined by performing 1,000 permutations for each trait [18]. For significant QTLs, 95% CI was defined by a decline of 1.5 LOD. After single QTL scans, pairwise evaluations for potential interactions between loci were made. At this stage, threshold LOD scores were strictly based on those recommended by Broman and Sen [16].

To determine whether the effect of QTL was specifically identified in the presence of the *A*^*y*^ allele, data for BC *A*^*y*^ and BC non-*A*^*y*^ mice were combined and reanalyzed using the agouti locus genotype as a covariate. Because the difference between the LOD score with the agouti locus genotype as an interactive covariate and the LOD score with the agouti locus genotype as an additive covariate concerns the test of the QTL × agouti locus genotype interaction, this test was performed for combined BC mice.

After QTL mapping, we genotyped interferon activated gene 202B (*Ifi202b*) and zinc finger protein 69 (*Zfp69*) to test their suitability as candidate genes for the QTLs.

### Statistical analysis using a linear model and partition method

Body weights of BC *A*^*y*^ males at 25 weeks of age were used as the dependent variable in the linear model and partition method with JMP ver. 9.0.1 statistical software. PGLC, CHO, TG, and HMW adiponectin levels were independent variables and treated as continuous variables. The best-fit linear model was generated from a full model by reducing nonsignificant terms using stepwise procedures. In the partition method, PGLC levels were used as an independent variable. We stopped first or second splits because of the small sample size. The clusters split in the partition method were treated as independent nominal variables. Comparisons of the associations between the clusters and other dependent variables (e.g., body weight at 25 weeks, BMI, PGLC, CHO, TG, and HMW adiponectin levels) were made by an F*-*test, followed by Student’s or Welch’s test. P values <0.05 was considered statistically significant. The average body weights of BC *A*^*y*^ males clustered in the partition method were modeled from 5 to 25 weeks of age using model 3 described by Eq. 3.

## Results

### Growth of DDDYM mice stops between 16 and 17 weeks, following which body weight gradually declines

**Figure 1:**
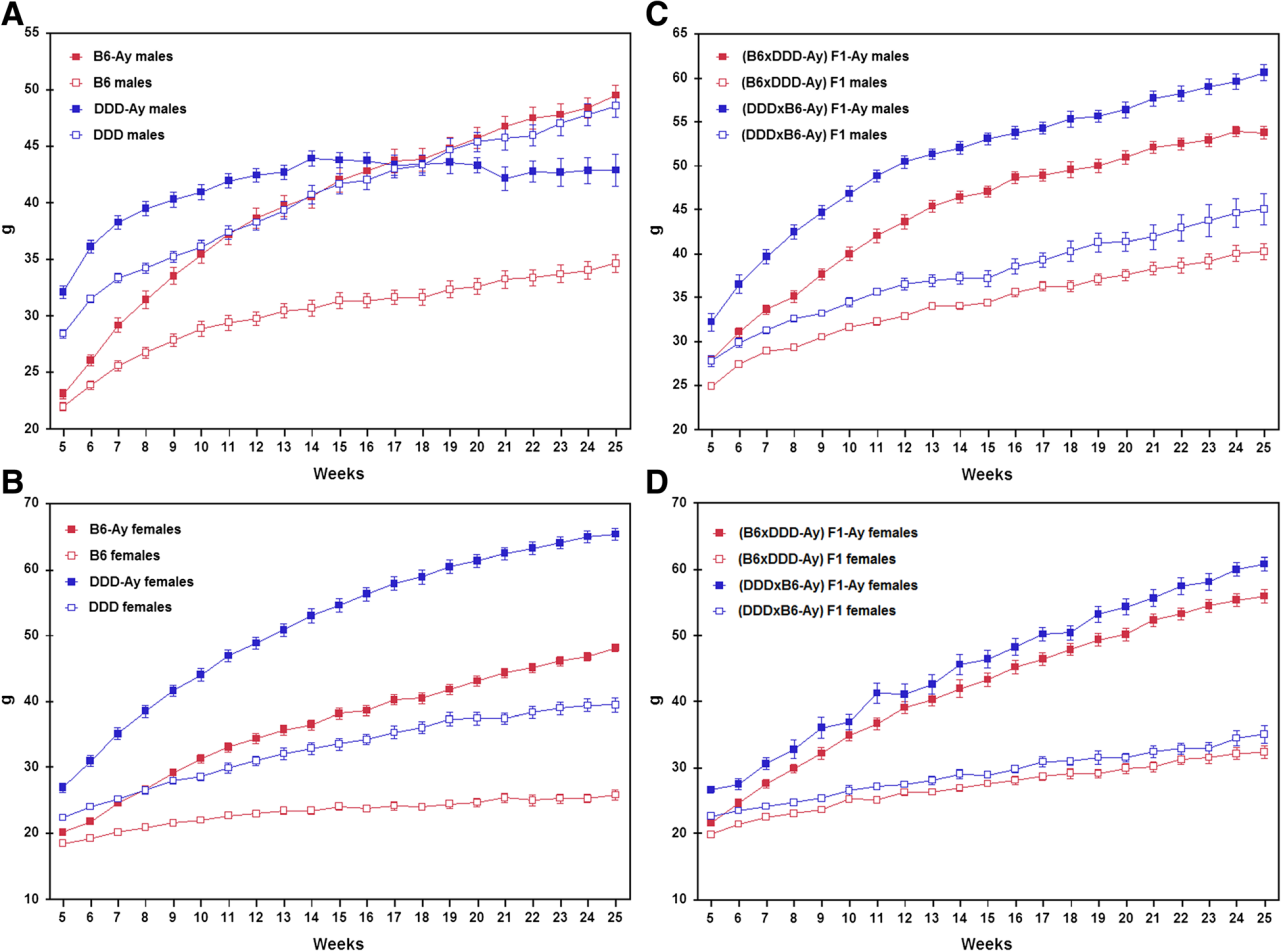
**Comparison of growth curves among group-housed inbred males (A) and females (B) and F**_**1 **_**males (C) and females (D).** Body weights were determined from 5 to 25 weeks of age. Each point represents the average body weight of each strain, and standard errors are indicated by error bars.

Figure 1 shows the growth curves of the parental inbred mouse strains (A, males; B, females) and F_1_ mice (C, males; D, females) from 5 to 25 weeks of age. The corresponding growth curve analytic results are shown in Table 3. Growth curve data were best fit to model 3 for 10 of 16 mouse categories: B6AF, B6AM, B6YF, B6YM, BDYF, BDYM, DBYM, DDDAF, DDDYF, and DDDYM. Growth curve data were best fit to model 2 for the remaining six categories: BDAF, BDAM, DBAF, DBAM, DBYF, and DDDAM. The fits of the observed data to all the three models were statistically significant (P < 0.05).

**Table 3:** Summary of growth models for mice categorized with respect to individual variables of strain*agouti*sex

		**Model 1**			**Model 2**				**Model 3**				
		**Values of parameters**			**Values of parameters**				**Values of parameters**				
Individual variable	Category	a	b	Residuals	a	b	Residuals	Adjusted R^2^	a	b	c	Residuals	Adjusted R^2^
	^a^B6AF	24.8	0.235	1.912	2.621	0.197	0.080	0.969	2.248	0.511	−0.063368	0.025	0.990
	^a^B6AM	33.3	0.200	1.938	2.751	0.249	0.124	0.959	2.176	0.732	−0.097586	0.029	0.990
	^a^B6YF	52.2	0.089	0.960	2.176	0.532	0.221	0.989	1.578	1.035	−0.101656	0.062	0.997
	^a^B6YM	50.9	0.118	0.476	2.509	0.443	0.256	0.973	1.593	1.212	−0.155501	0.016	0.998
	^b^BDAF	31.0	0.170	1.277	2.530	0.291	0.002	0.993	2.555	0.270	0.004	0.002	0.993
	^b^BDAM	38.6	0.180	1.537	2.805	0.276	0.061	0.992	2.686	0.376	−0.020256	0.053	0.992
Strain*Agouti*Sex	^a^BDYF	65.2	0.076	0.801	2.177	0.584	0.143	0.994	1.623	1.050	−0.094138	0.015	0.999
	^a^BDYM	55.3	0.131	0.655	2.742	0.400	0.208	0.975	1.973	1.047	−0.130541	0.023	0.997
	^b^DBAF	32.7	0.179	1.323	2.654	0.270	0.046	0.985	2.975	0.000	0.054	0.020	0.993
	^b^DBAM	42.8	0.174	1.386	2.882	0.282	0.029	0.991	2.943	0.232	0.010	0.028	0.990
	^b^DBYF	68.0	0.081	1.329	2.387	0.536	0.062	0.995	2.366	0.554	−0.003482	0.062	0.995
	^a^DBYM	60.0	0.151	0.770	2.998	0.352	0.203	0.959	2.119	1.090	−0.14911	0.022	0.995
	^a^DDDAF	39.7	0.137	1.217	2.523	0.364	0.024	0.997	2.407	0.462	−0.019735	0.019	0.998
	^b^DDDAM	46.9	0.160	1.600	2.875	0.312	0.042	0.994	2.800	0.375	−0.012714	0.040	0.994
	^a^DDDYF	72.3	0.094	1.165	2.523	0.534	0.691	0.976	1.453	1.433	−0.1816	0.000	1.000
	^a^DDDYM	43.4	0.292	2.044	3.375	0.133	0.517	0.670	2.226	1.098	−0.194955	0.035	0.976

^a^Model 3 best fits the observed data.

^b^Model 2 best fits the observed data.

**Figure 2:**
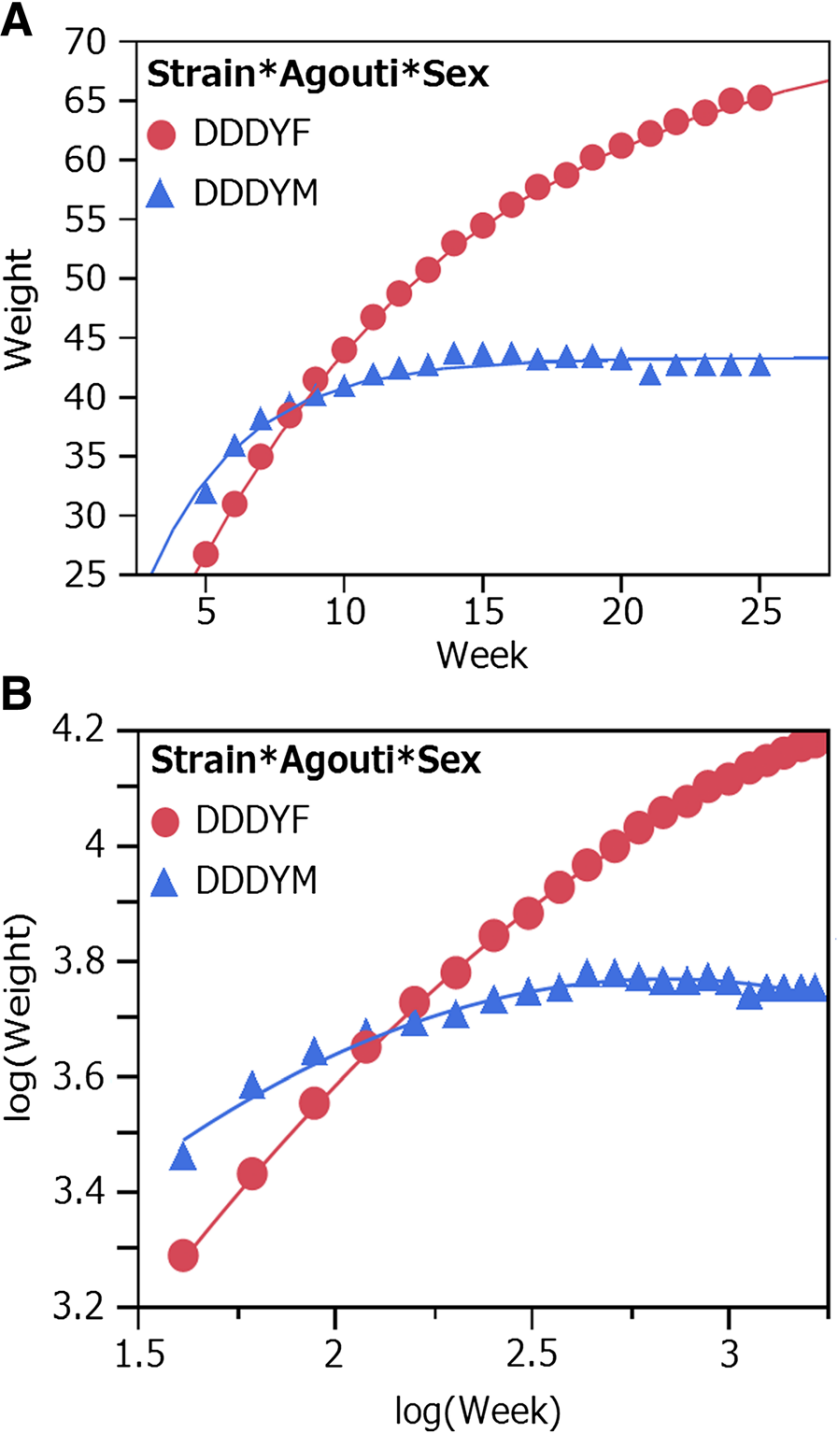
**Growth curves of DDDYF and DDDYM mice based on Model 1 (A) and Model 3 (B).** Lines indicate predicted values and markers indicate observed data.

In model 1, the maximum body weight ranged from 24.8 g (B6AF mice) to 72.3 g (DDDYF mice). In non-*A*^*y*^ mice, the maximum body weight of males was greater than that of females within the same strain. In contrast, in *A*^*y*^ mice, the maximum body weight of females was greater than that of males within the same strain. The growth rate was the greatest in DDDYM mice (0.292). The body weight of DDDYM mice reached the maximum value at the earliest age. The difference in the maximum body weight between males and females was the greatest in DDDY mice (28.9 g) (Figure 2A).

In accordance with the results with model 1, the growth rate was the lowest in DDDYM mice (0.133) in model 2. The adjusted R-squared value was the smallest in DDDYM mice (0.670). The adjusted R-squared values for the other categories were >0.900.

In model 3, DDDYM mice reached their maximum body weights (mean = 43.5 g) at 16.7 weeks of age (Figure 2B). The maximum body weight of DDDYM mice was nearly equal to that obtained in model 1 (mean = 43.4 g). The body weights of the other mouse categories did not reach the maximum values during the observation period. The values for the parameter *c* were positive in BDAF, DBAF, and DBAM mice, but were negative in the remaining 13 categories. The smallest value for the parameter *c* was found in DDDYM mice (−0.195).

### Blood phenotypes of DDD-*A*^*y*^ males

**Figure 3:**
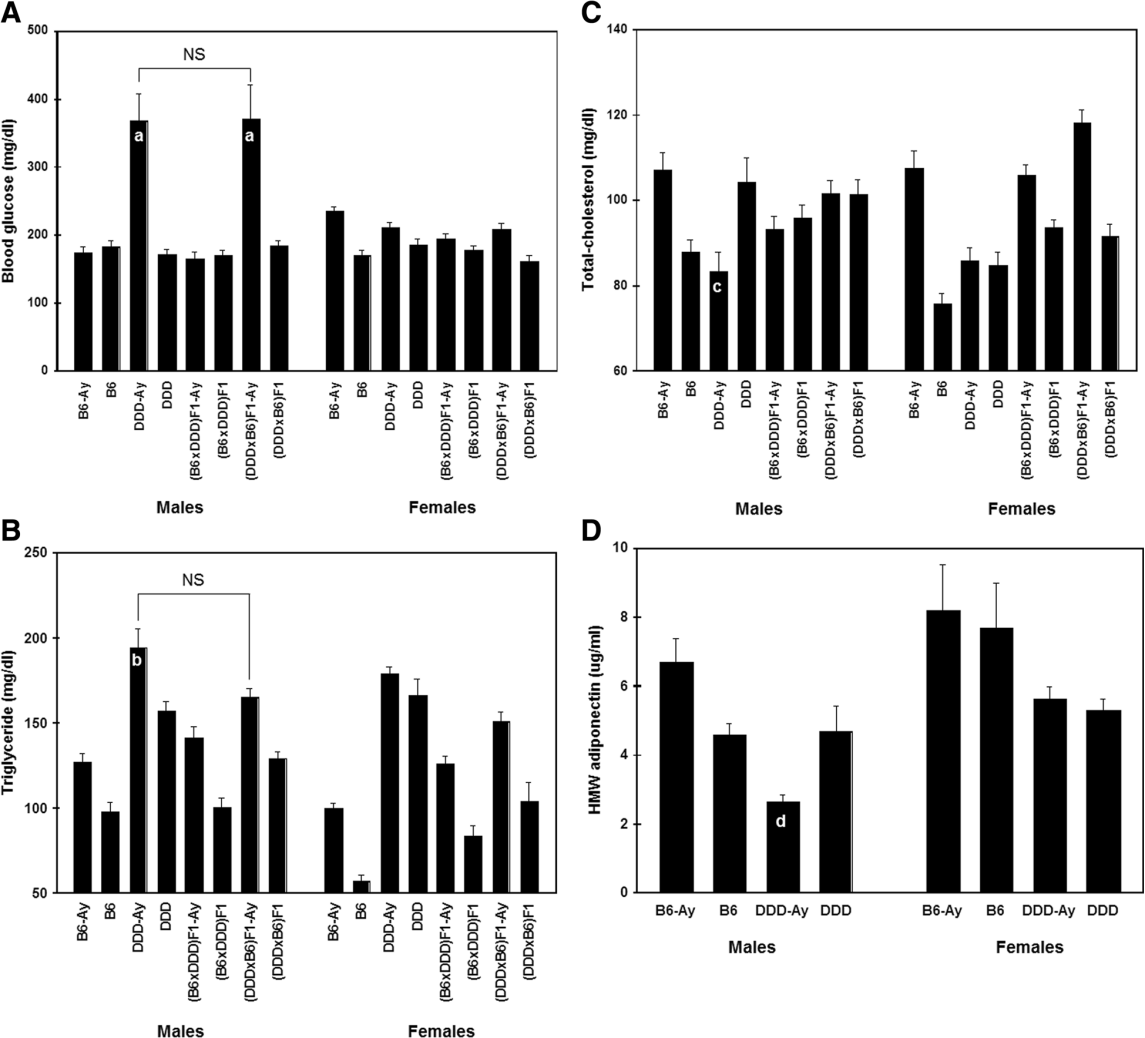
**Comparison of blood phenotypes at 25 weeks of age after 4 h of fasting in parental and F**_**1 **_**mouse strains.** (**A**) Blood glucose levels, (**B**) plasma triglyceride levels, (**C**) plasma total-cholesterol levels, and (**D**) plasma HMW adiponectin levels. Error bars indicate standard errors. The sample sizes of mice for glucose, triglyceride, and total-cholesterol determination was the same as those in Table 1. HMW adiponectin levels were determined in 7–10 mice for each strain. Males and females were analyzed separately. All pairs were compared by Tukey–Kramer HSD tests; however, the results of statistical comparisons are shown only for those related to DDD-*A*^*y*^ males. **A**, Significant difference (P <0.0001) versus DDD, B6-*A*^*y*^, B6, (DDD × B6) F_1_, (B6 × DDD) F_1_-*A*^*y*^, and (B6 × DDD) F_1_. NS, no significant difference between DDD-*A*^*y*^ and (DDD × B6) F_1_-*A*^*y*^. **B**, Significant difference (P <0.0001) versus B6-*A*^*y*^, B6, (DDD × B6) F_1_, (B6 × DDD) F_1_-*A*^*y*^, and (B6 × DDD) F_1_, and significant difference (P <0.002) versus DDD. NS, no significant difference between DDD-*A*^*y*^ and (DDD × B6) F_1_-*A*^*y*^. **C**, Significant difference (P <0.01) versus DDD and B6-*A*^*y*^. **D**, Significant difference (P <0.0001) versus B6-*A*^*y*^, and significant difference (P <0.05) versus DDD and B6.

We determined the BGLC, CHO, TG, and HMW adiponectin levels in parental strains at 25 weeks of age. The first three levels were also determined for F_1_ mice. DDD-*A*^*y*^ and DB F_1_-*A*^*y*^ males had significantly higher BGLC levels than mice with other genotypes. No significant differences in BGLC levels were observed between DDD-*A*^*y*^ and DB F_1_-*A*^*y*^ males (Figure 3A). DDD-*A*^*y*^ and DDD mice clearly had higher TG levels than B6-*A*^*y*^ and B6 mice for both the sexes (Figure 3B). In particular, DDD-*A*^*y*^ males had significantly higher TG levels than the other strains, except for DB F_1_-*A*^*y*^ males. In contrast, DDD-*A*^*y*^ and DDD mice were not hypercholesterolemic compared with B6-*A*^*y*^ and B6 mice, respectively, for both the sexes (Figure 3C). Conversely, DDD-*A*^*y*^ males had significantly lower CHO levels than DDD and B6-*A*^*y*^ males. DDD-*A*^*y*^ males had significantly lower HMW adiponectin levels than B6-*A*^*y*^, B6, and DDD males (Figure 3D).

### Histology

No marked abnormalities were observed in the pancreas and adipose tissues of any of the mice. Liver lesions were observed only in *A*^*y*^ mice, although these were found in both the sexes. Indeed, fatty changes were observed in DDD-*A*^*y*^ and B6-*A*^*y*^ males, while glycogen deposition was observed in DDD-*A*^*y*^ and B6-*A*^*y*^ females. Deposition of large fat droplets was observed in DDD-*A*^*y*^ and B6-*A*^*y*^ females (data not shown).

### QTLs associated with lower body weight of DDD.Cg-*A*^*y*^ males are identified on chromosomes 1 and 4

We conducted QTL analyses for body weight (from 5 to 25 weeks of age), BMI, and BGLC, PGLC, CHO, TG, and HMW adiponectin levels using the results of 100 BC *A*^*y*^ males. Seven BC *A*^*y*^ males had BGLC levels of >600 mg/dl, the upper limit of detection for the method used. Their BGLC levels were assumed to be 600 mg/dl. To make glucose determination more exact, we also measured PGLC levels; PGLC levels were highly correlated with BGLC levels (*r* = 0.95; P <0.0001). Phenotypes are shown in Table 4 along with those for 96 BC non-*A*^*y*^ males. Phenotypic values were significantly greater in BC *A*^*y*^ males than in BC non-*A*^*y*^ males (PGLC and HMW adiponectin levels were not determined in BC non-*A*^*y*^ males).

**Table 4:** Phenotypes of BC males

**Phenotype at 25 weeks**	**Mean ± SE values (range)**	
	**BC non-*A***^***y ***^**males (n = 96)**	**BC *A***^***y ***^**males (n = 100)**
Body weight (g)	43.64 ± 0.59	49.37 ± 0.51
	(33.05–57.90)	(36.13–60.28)
BMI	4.00 ± 0.04	4.48 ± 0.04
	(3.22–4.94)	(3.61–5.31)
Blood glucose (mg/dl) ^a^	209.1 ± 4.4	329.7 ± 14.9
	(115–379)	(110–600)
Plasma glucose (mg/dl)	nd	427.3 ± 18.6
		(164–869)
Total-cholesterol (mg/dl)	121.7 ± 2.7	154.0 ± 4.4
	(69–198)	(74–303)
Triglyceride (mg/dl)	245.2 ± 9.9	622.0 ± 24.7
	(98–778)	(109–1,247)
HMW adiponectin (μg/ml)	nd	3.81 ± 0.13
		(1.67–9.15)

^a^Seven mice had blood glucose levels that exceeded the upper limit of the measuring method (600 mg/dl). Therefore, mean ± SE values were calculated by assuming that their blood glucose levels were 600 mg/dl.

Values are significantly greater in BC *A*^*y*^ than in BC non-*A*^*y*^ males. nd, not determined.

**Figure 4:**
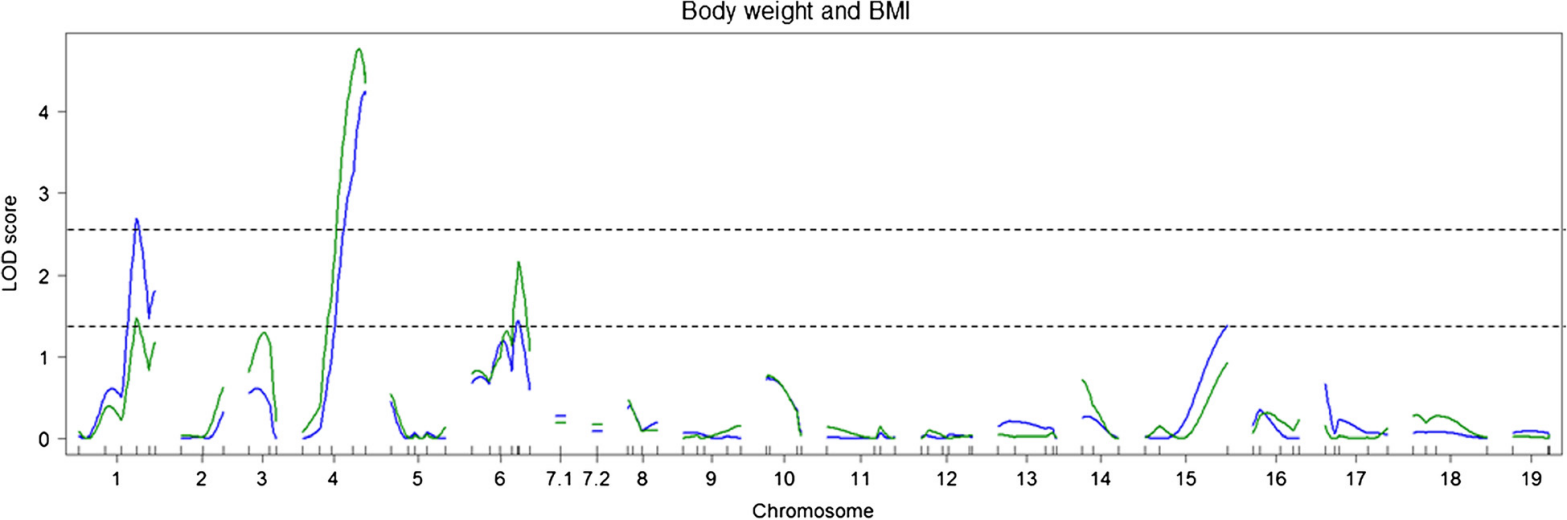
**Genome-wide LOD score plots for body weights at 25 weeks of age (blue lines) and BMI (green lines).** Horizontal dashed lines indicate significant and suggestive threshold LOD scores determined by 1,000 permutations.

**Figure 5:**
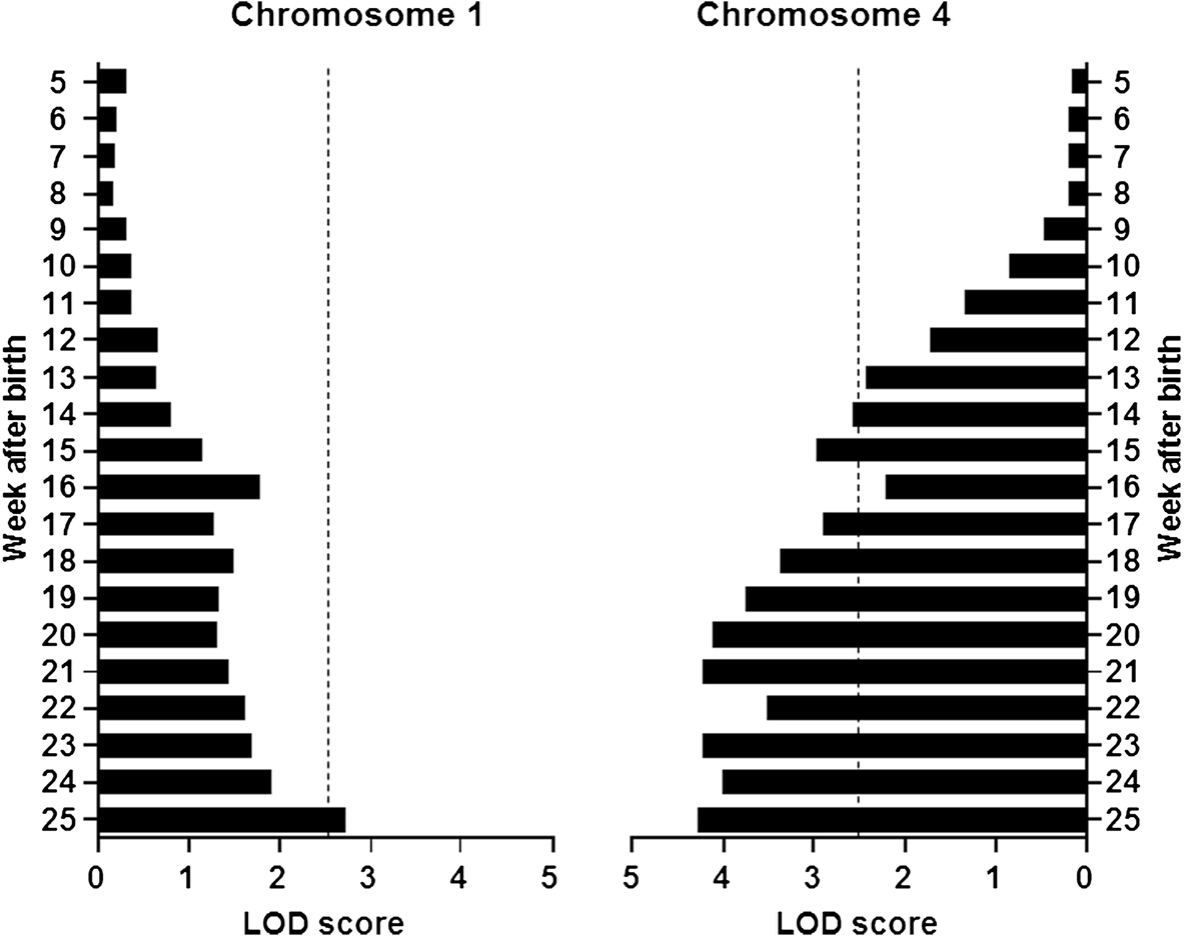
Changes in LOD scores for QTLs on chromosomes 1 and 4 from 5 to 25 weeks of age.

**Figure 6:**
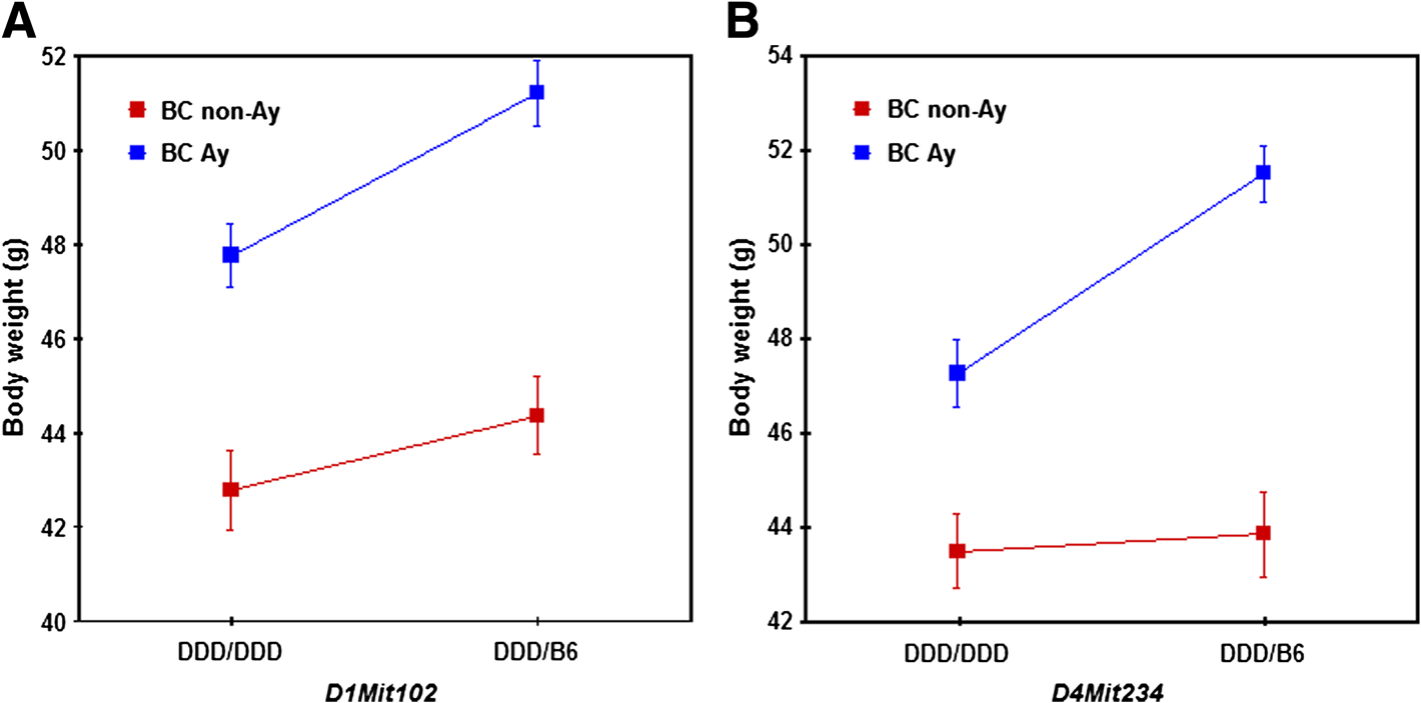
**Allele effects of QTLs on chromosomes 1 (A) and 4 (B) on body weight at 25 weeks of age.** The DDD allele was associated with lower body weight at both loci in BC *A*^*y*^ mice. In particular, the allele effect of the QTL differed between BC *A*^*y*^ and BC non-*A*^*y*^ mice.

The results of single QTL scans are summarized in Table 5. For the body weight at 25 weeks of age, two significant QTLs were identified on chromosomes 1 and 4 (Figure 4). For 21 QTL analyses of body weight conducted weekly (from 5 to 25 weeks), significant QTLs were identified only on chromosomes 1 and 4. QTL results for chromosome 1 were significant only for body weight at 25 weeks of age (Figure 5). In contrast, QTL results for chromosome 4 were significant from 14 weeks onward, except at 16 weeks. For both QTLs, the DDD allele was associated with a lower body weight (Figure 6).

**Table 5:** **Summary of single QTL scans in BC-*A***^***y ***^**mice**

**Trait**	**Chromosome**	**Location (cM)**^**a**^	**95% CI (cM)**^**b**^	**Max LOD**^**c**^	**Nearest marker**	**High allele**^**d**^
Body weight at 25 weeks	1	56	45–73	2.69*	*D1Mit102*	B6
	4	61	41–61	4.25*	*D4Mit234*	B6
	6	44	0–56	1.44	*D6Mit108*	B6
	15	80	0–80	1.39	*D15Mit193*	DDD
BMI	1	56	0–73	1.48	*D1Mit102*	B6
	4	55	36–61	4.77*	*D4Mit234*	B6
	6	45	0–56	2.16	*D6Mit256*	B6
Blood glucose	4	61	21–61	1.88	*D4Mit234*	DDD
	7.1	0	na	1.54	*D7Mit250*	B6
	12	30	0–49	1.82	*D12Mit259*	B6
Plasma glucose	4	61	13–65	1.55	*D4Mit234*	DDD
	7.1	0	na	1.56	*D7Mit250*	B6
	12	30	10–49	2.28	*D12Mit259*	B6
	19	11	0–35	1.73	*D19Mit32*	DDD
Plasma triglyceride	8	14	0–28	1.77	*D8Mit249*	DDD
	12	27	7–44	2.39	*D12Mit259*	DDD
	18	0	0–71	1.42	*D18Mit21*	DDD
HMW adiponectin	4	39	10–61	1.89	*D4Mit12*	B6
	7.1	0	na	1.57	*D7Mit250*	DDD

^a^Location indicates a chromosomal position showing a peak LOD score in cM.

^b^95% CI is defined by a 1.5-LOD support interval. na, not available.

^c^Maximum LOD score for QTL. Significant QTLs are indicated by * (suggestive QTLs are shown without asterisks).

^d^Allele associated with higher trait values.

**Figure 7:**
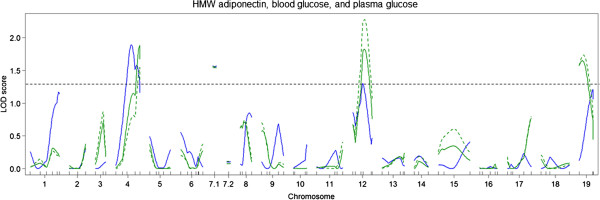
**Genome-wide LOD score plots for plasma HMW adiponectin levels (blue lines), blood glucose levels (solid green lines), and plasma glucose levels (dashed green lines).** A horizontal dashed line indicates a suggestive threshold LOD score determined by 1,000 permutations.

**Figure 8:**
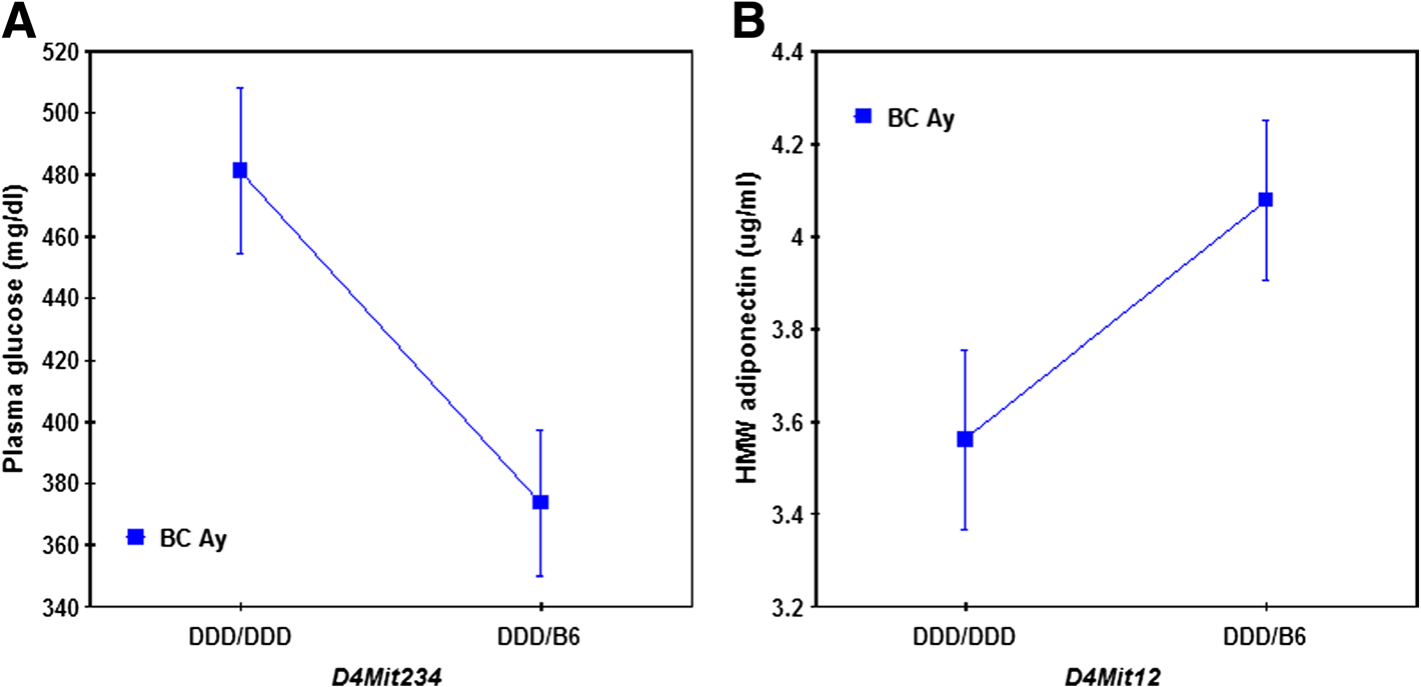
**Allele effects of a QTL on chromosome 4 on (A) plasma glucose and (B) plasma HMW adiponectin levels in BC *A***^***y***^**mice.** The DDD allele was associated with increased plasma glucose levels and decreased HMW adiponectin levels.

**Figure 9:**
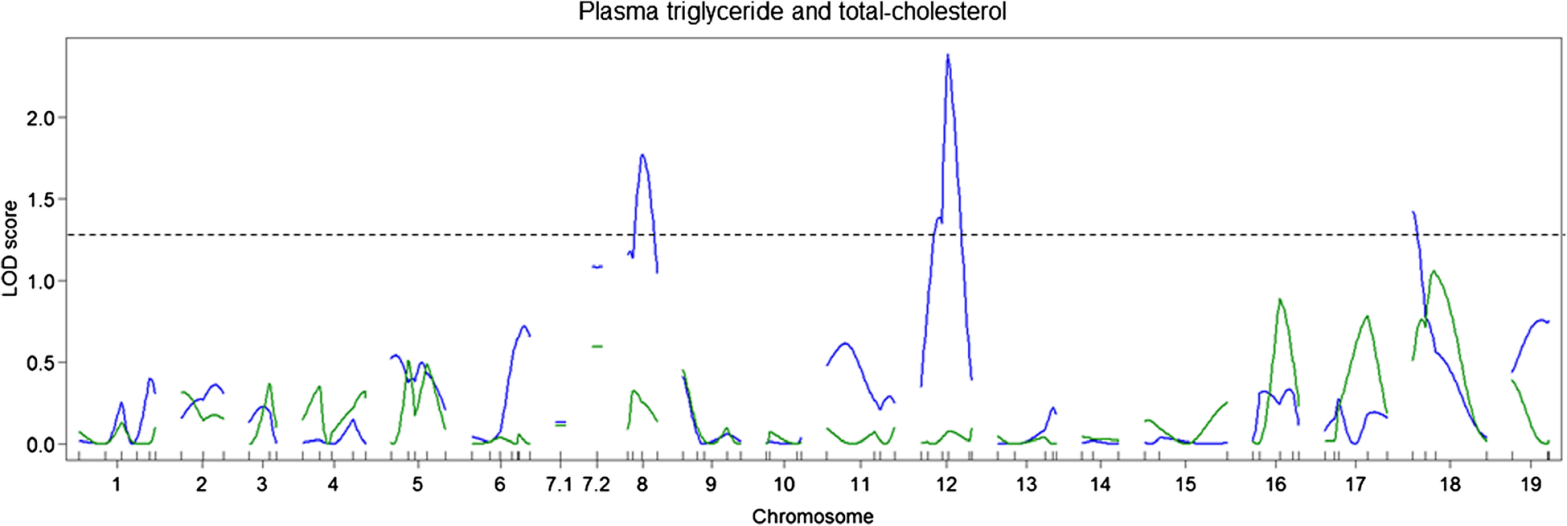
**Genome-wide LOD score plots for plasma triglyceride (blue lines) and total-cholesterol (green lines) levels.** A horizontal dashed line indicates a suggestive threshold LOD score determined by 1,000 permutations.

For BMI, one significant QTL was identified on chromosome 4 (Figure 4). For this QTL, the DDD allele was associated with lower BMI. Suggestive QTLs for HMW adiponectin, BGLC, and PGLC levels were colocalized with the QTL for body weight on chromosome 4 but not with the QTL on chromosome 1 (Figure 7). The DDD allele was associated with increased BGLC and PGLC levels and decreased HMW adiponectin levels (Figure 8). For BGLC and PGLC, suggestive QTLs were also identified on chromosomes 7.1 and 12. At both loci, the B6 allele was associated with increased glucose levels. Three suggestive QTLs were identified for TG levels on chromosomes 8, 12, and 18 (Figure 9). The suggestive QTL on chromosome 12 coincided with the suggestive QTLs for BGLC and PGLC levels. The DDD allele was associated with increased TG levels. No significant or suggestive QTLs were identified for CHO levels. The effects of the loci on chromosomes 1 and 4 for TG and CHO levels were negligible.

**Figure 10:**
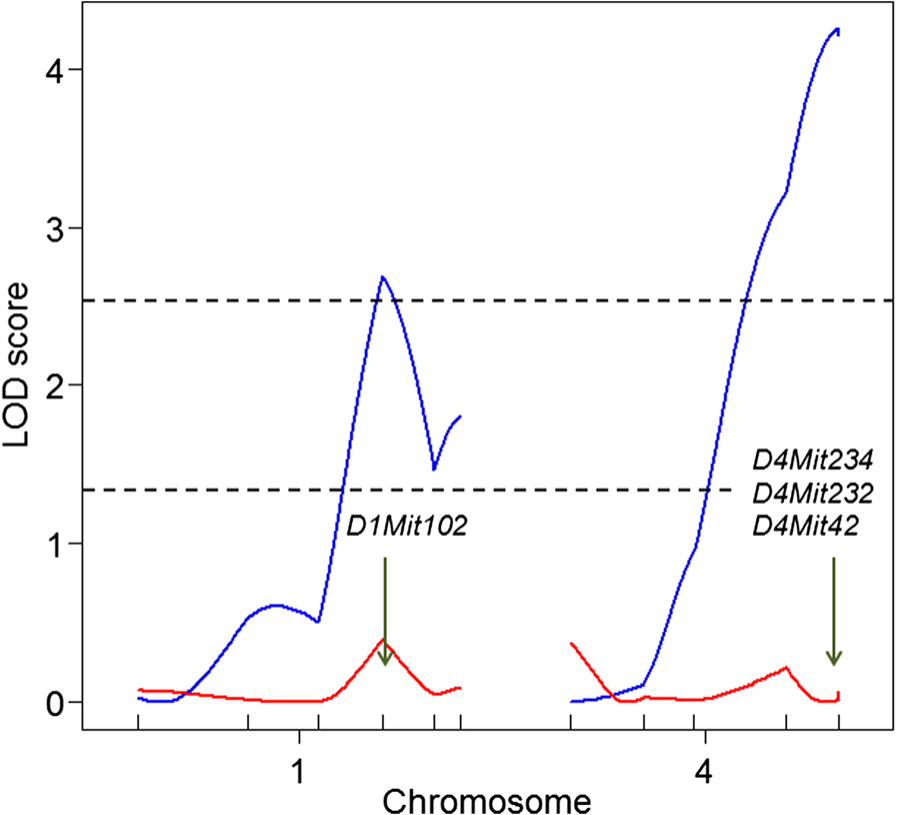
**LOD scores for QTLs on chromosomes 1 and 4 for body weight at 25 weeks of age in BC *A***^***y ***^**(blue lines) and BC non-*A***^***y ***^**males (red lines).** QTL peak is indicated by an arrow. The nearest markers are also shown.

**Figure 11:**
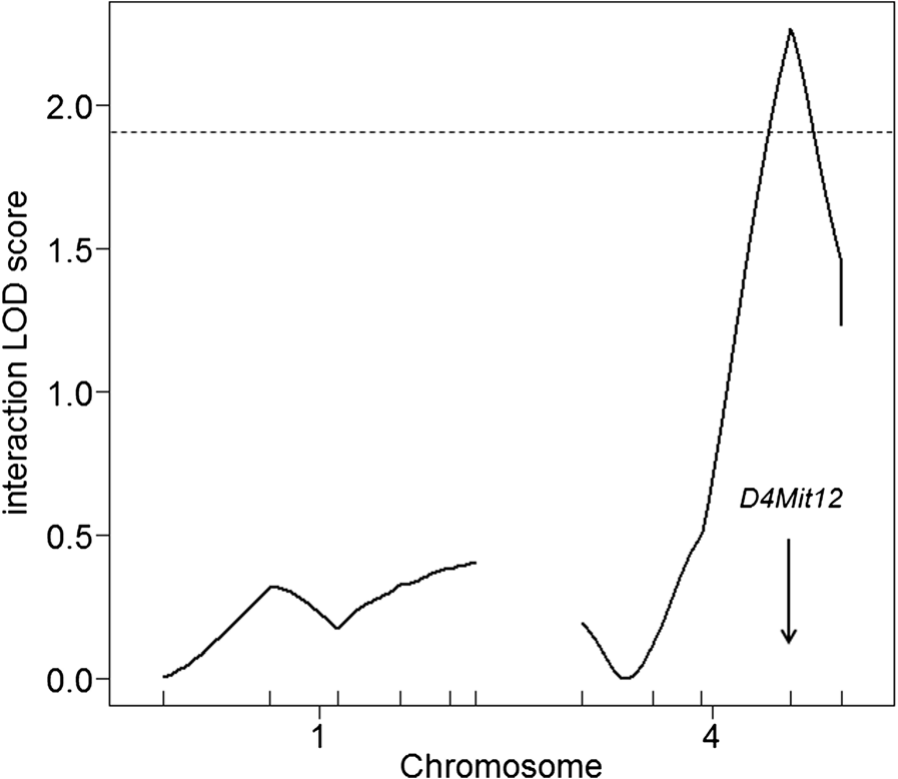
**Evidence for and the chromosomal location of a QTL that interacts with the *A***^***y***^**allele on chromosome 4.** Nearest marker for the QTL peak is indicated by an arrow.

We next addressed whether the effects of QTLs on chromosomes 1 and 4 could be identified in the absence of the *A*^*y*^ allele (i.e., in BC non-*A*^*y*^ males), i.e., whether or not QTLs on chromosomes 1 and 4 interacted with the *A*^*y*^ allele. When QTL analysis of body weight at 25 weeks of age was conducted for 96 BC non-*A*^*y*^ males, there was no evidence of linkage with regard to chromosomes 1 and 4 (Figure 10). When BC *A*^*y*^ and BC non-*A*^*y*^ males were combined and reanalyzed using the agouti locus genotype (*A*^*y*^ or non-*A*^*y*^) as a covariate, significant evidence of a QTL × covariate interaction was identified for the QTL on chromosome 4 but not for the QTL on chromosome 1 (Table 6 and Figure 11). Thus, the effect of the QTL on chromosome 4 differed between in BC *A*^*y*^ and in BC non-*A*^*y*^ males, i.e., the effect of this QTL differed between in the presence and absence of the *A*^*y*^ allele (Figure 6B).

**Table 6:** **Single QTL scans for body weight at 25 weeks in combined BC mice using the *agouti *genotype as a covariate**

**Chromosome**	**LOD scores (peak position, cM)**		
	***Agouti *as an additive covariate (LOD**_**a**_**)**^**a**^	***Agouti *as an interactive covariate (LOD**_**f**_**)**^**b**^	**LOD**_**i **_**(LOD**_**f**_**-LOD**_**a**_**)**^**c**^
4	None	3.52 (61)	2.27 (49)

Only significant QTLs are listed.

^a^Significant threshold LOD score was 2.57.

^b^Significant threshold LOD score was 3.28.

^c^LOD_i_ is the difference between the LOD score with *agouti* as an interactive covariate (LOD_f_) and the LOD score with *agouti* as an additive covariate (LOD_a_). This derives from a test of the QTL × *agouti* interaction. Significant threshold LOD score was 1.91.

We further mapped these QTLs using a reference map position retrieved from Mouse Genome Informatics (MGI, http://www.informatics.jax.org/) in order to search possible candidate genes for the QTLs. For this purpose, we excluded genotype data on *D1Mit16* and *D4Mit234* because information on Mbp position was not available for these markers. Instead, we newly genotyped *D1Mit36* (169.21 Mbp), *D1Mit356* (172.89 Mbp), *D1Mit155* (194.43 Mbp), and *D4Mit204* (133.43 Mbp). Results of single QTL scans for body weight at 25 weeks are summarized in Table 7. The 95% CI for QTLs on chromosomes 1 and 4 contained plausible candidate genes, *Ifi202b* and *Zfp69*, respectively, both of which were known to be related with obesity-associated diabetes [19,20].

**Table 7:** Mapping of QTLs on chromosomes 1 and 4 for body weight at 25 weeks using reference map position

**Chromosome**	**Location (Mbp)**^**a**^	**95% CI (Mbp)**^**b**^	**Max LOD**^**c**^	**Candidate gene (Position, Mbp)**
1	147.25	106.49–188.44	2.69	*Ifi202b* (173.96)
4	145.06	112.44–151.57	4.25	*Zfp69* (120.93)

^a^Location indicates a chromosomal position showing a peak LOD score in Mbp.

^b^95% CI is defined by a 1.5-LOD support interval in Mbp.

^c^Maximum LOD score for QTL.

**Figure 12:**
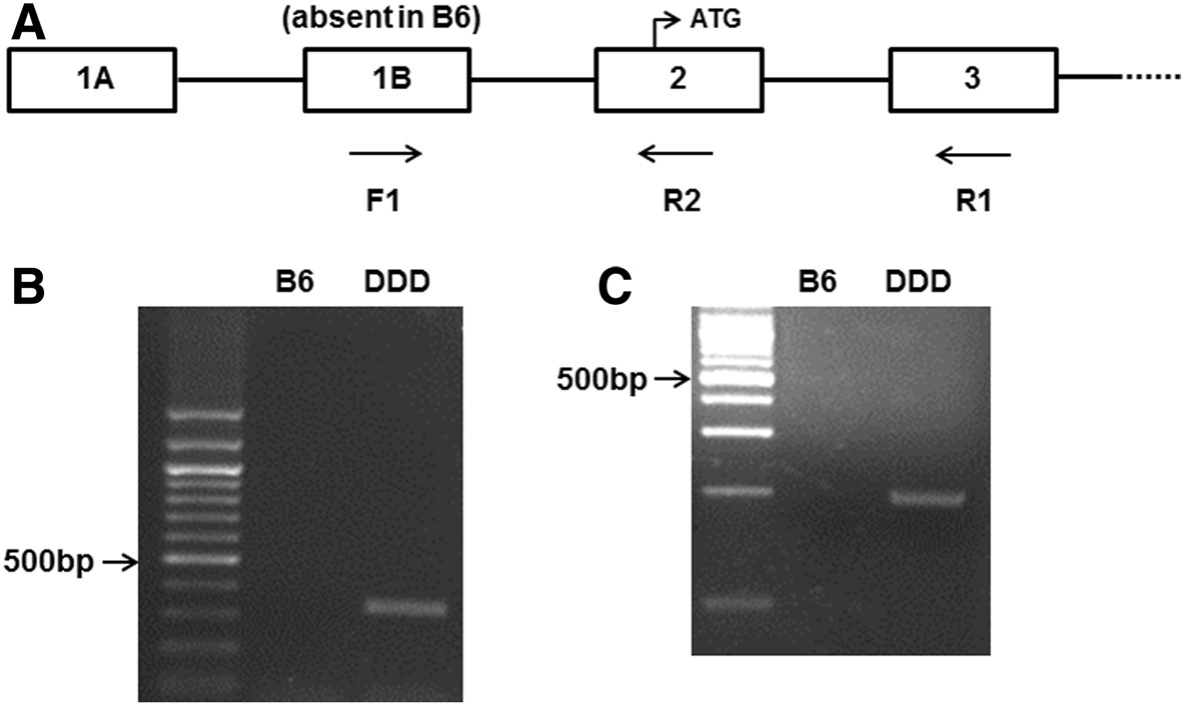
**Genotyping of *Ifi202b *in B6 and DDD mice.** (**A**) Genomic organization and PCR primers used for genotyping of *Ifi202b* on chromosome 1 based on reference [19]. (**B, C**) Identification of the cDNA variants in B6 and DDD by PCR. 100 bp ladder markers are shown in the left.

**Figure 13:**
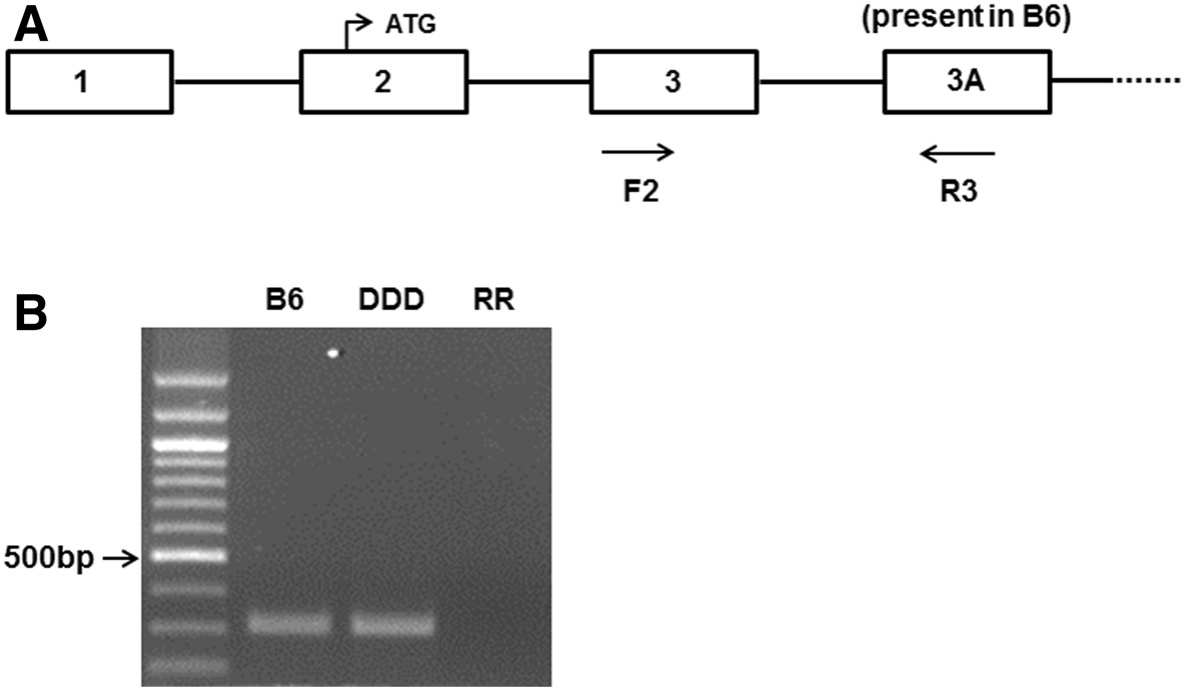
**Genotyping of *Zfp69 *in B6 and DDD mice.** (**A**) Genomic organization and PCR primers used for genotyping of *Zfp69* on chromosome 4 based on reference [20]. (**B**) Identification of the cDNA variants in B6, DDD, and unrelated RR by PCR. 100 bp ladder markers are shown in the left.

Therefore, we genotyped *Ifi202b* and *Zfp69* to test their suitability as candidate genes for the QTLs. For the genotyping of *Ifi202b*, we performed two kinds of PCR using cDNA as a template [19]. As shown in Figure 12A and Table 8, forward primer corresponding to the corresponding to the exon 1B (F1), which was missing in B6, in combination with reverse primers on exon 3 (R1) and 2 (R2) amplified a PCR product only on DDD cDNA (Figure 12B and Figure 12C, respectively). For the genotyping of *Zfp69*, we performed PCR using cDNA as a template [20]. As shown in Figure 13A and Table 8, forward primer corresponding to the exon 3 (F2) in combination with reverse primer on B6-specific exon 3A (R3) amplified a PCR products on B6 and DDD (Figure 13B). We also performed PCR using unrelated RR/Sgn strain but it did not generate a PCR product.

**Table 8:** A list of primers used for genotyping of candidate genes

**Primer name**^**a**^	**Primer sequence (5’-to-3’)**
F1	CCC TCT TCC TTT ACA CCC AAC
F2	CCA TGA TGC TAA TGG GAC AC
R1	TTG GGG TGT GTG ACT TTT TG
R2	GCC TGG GAC AGA TGT CTC TT
R3	TCT ACG GCA AAG CTT TAT TGC

^a^Primer name beginning with “F” means that the primer was used as a forward primer, and that beginning with “R” means that the primer was used as a reverse primer.

### Statistical analysis with a linear model and partition method

The best-fit linear model was as follows:

(4) $$\begin{array}{l} \mathrm{Body}\;\mathrm{weight}=a+b\times \left(\mathrm{PGLC}\;\mathrm{levels}\right)+c\\ \;\times \left(\mathrm{HMW}\;\mathrm{adiponectin}\;\mathrm{levels}\right)+d\\ \;\times {\left(\mathrm{PGLC}\;\mathrm{levels}\right)}^{2}+\epsilon \end{array}$$

**Figure 14:**
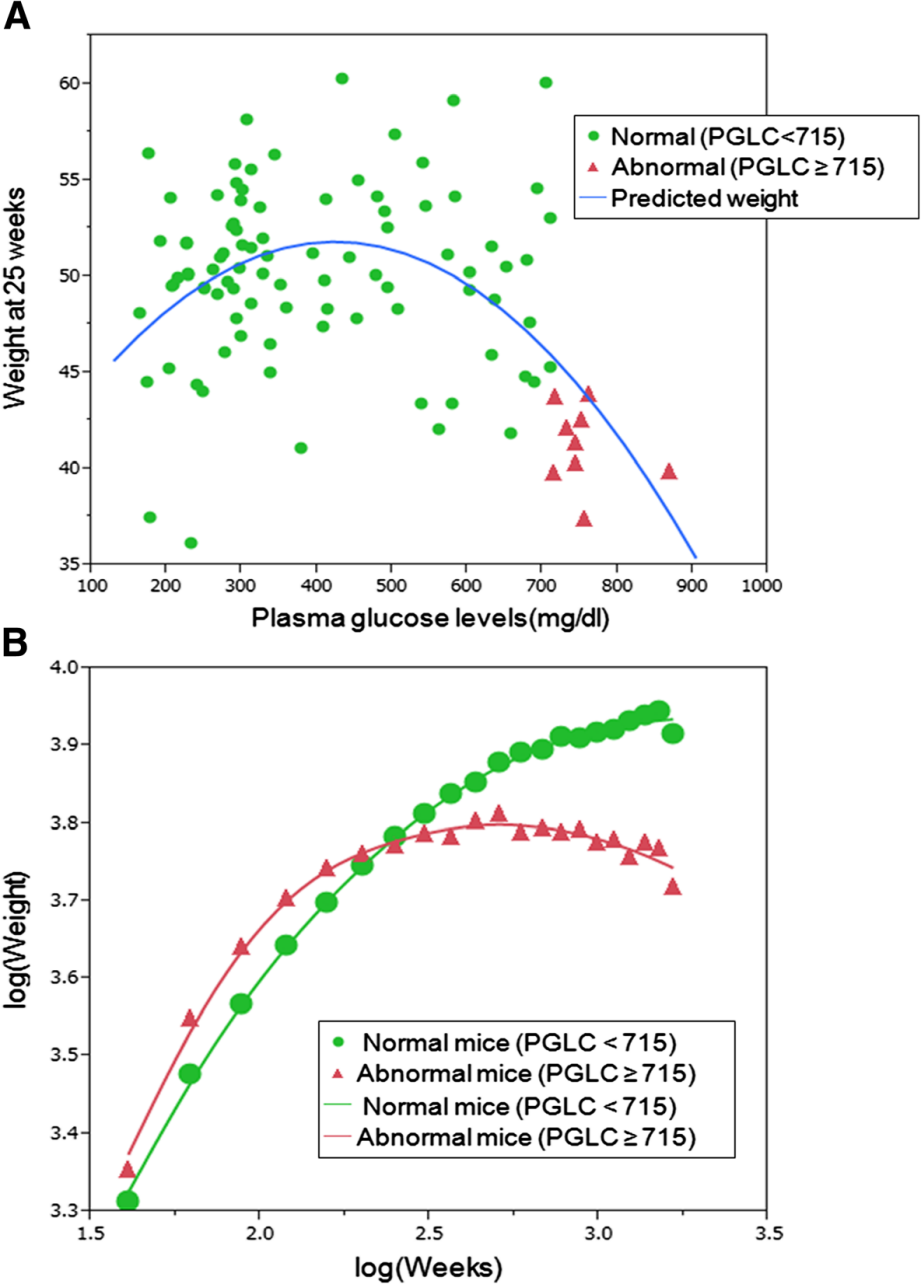
**Statistical analysis with a linear model and partition method.** (**A**) Body weights of BC *A*^*y*^ males at 25 weeks of age versus PGLC levels. BC *A*^*y*^ males were categorized into two groups based on a partition method. The first group included nine abnormal mice and the second group included 91 normal mice. PGLC levels were ≥715 mg/dl in abnormal mice and ≤715 mg/dl in normal mice. (**B**) Growth curves of abnormal and normal BC *A*^*y*^ males based on model 3. Lines indicate predicted values and markers indicate observed data.

In Eq. 4, PGLC and HMW adiponectin levels were linearly related to the body weight and the square of PGLC levels was related to the body weight. Estimates for the four parameters were as follows: *a* = 32.4, *b* =0.068, *c* = 1.06, and *d* = −7.5×10^−5^. The adjusted R-squared value and the root mean square error were 0.255 and 4.37 g, respectively. Body weight at 25 weeks of age increased in direct proportion to HMW adiponectin levels. Figure 14A shows the association between the observed body weights at 25 weeks of age and the predicted body weights from Eq. 4. The predicted body weight reached a maximum (51.9 g) at the point when the PGLC level was 427.3 mg/dl.

When the body weight at 25 weeks of age was used as the dependent variable, BC *A*^*y*^ males were categorized into two groups with the first split of the partition method. The first group included nine mice with PGLC levels ≥715 mg/dl, and the second group included 91 mice with PGLC levels <715 mg/dl. The average body weight of the first group was 41.2 g and that of the second group was 50.2 g. We defined the first group as “abnormal mice” and the second group as “normal mice.” Abnormal mice were characterized by higher PGLC levels and lower HMW adiponectin levels compared with normal mice.

In Figure 14A, the nine abnormal mice were mapped to the lower right of the plot area, in which mice had PGLC levels ≥715 mg/dl and body weights ranging from 37.4 g to 43.8 g. Comparisons of the phenotypes between these two groups are summarized in Table 9. Body weight, BMI, BGLC levels, PGLC levels, and HMW adiponectin levels significantly differed between these abnormal and normal mice. In contrast, CHO and TG levels did not differ significantly between these two groups.

**Table 9:** **Comparison of phenotypes between two groups of BC *A***^***y ***^**males clustered by the partition method**

**Phenotypes at 25 weeks**	**Mean ± SE values (95% confident interval)**	
	**Abnormal mice (PGLC ≥ 715)**^**b**^	**Normal mice (PGLC < 715)**
	**(n = 9)**	**(n = 91)**
Body weight (g)	41.2 ± 0.70^c^	50.17 ± 0.47
	(39.59–42.83)	(49.23–51.12)
BMI	3.90 ± 0.10^c^	4.54 ± 0.03
	(3.70–4.10)	(4.48–4.60)
Blood glucose (mg/dl)^a^	592.2 ± 4.6^c^	303.7 ± 13.7
	(581.5–602.9)	(276.6–330.8)
Plasma glucose (mg/dl)	754.8 ± 15.3^c^	394.9 ± 16.9
	(719.6-790.0)	(361.3–428.5)
Total-cholesterol (mg/dl)	133.4 ± 14.7	156.0 ± 4.6
	(104.3–162.6)	(146.8–165.2)
Triglyceride (mg/dl)	734.1 ± 81.7	610.9 ± 25.7
	(571.9–896.3)	(560.0–661.9)
HMW adiponectin (μg/ml)	2.61 ± 0.13^c^	3.93 ± 0.14
	(2.29–2.92)	(3.66–4.21)

^a^Seven mice had blood glucose levels that exceeded the upper limit of the measuring method (600 mg/dl). Therefore, mean ± SE values were calculated by assuming that their blood glucose levels were 600 mg/dl.

^b^PGLC indicates plasma glucose levels.

^c^Values are significantly different between abnormal mice and normal mice.

Figure 14B shows a comparison of the growth curves of abnormal and normal mice using model 3. For abnormal mice, estimates for the three parameters in this model were *a* = 1.33, *b* = 1.82, and *c* = −0.34. For normal mice, these estimates were *a* = 1.45, *b* = 1.56, and *c* = −0.25. The adjusted R-squared value and the root mean square errors were 0.99 and 0.016, respectively. The average body weight of abnormal mice was greater than that of normal mice until 10.7 weeks. Following this, body weight trends reversed between these two groups; normal mice became heavier than abnormal mice. Abnormal mice reached their maximum body weight (45.2 g) at 15.1 weeks of age, following which their body weight declined.

## Discussion

### Growth of DDDYM mice was peculiar among the 16 categories, and the lower body weight of the DDDYM mice was a consequence of body weight loss

From the results of model 1, the maximum body weight of males was greater than that of females of the same strain in non-*A*^*y*^ mice (Table 3). The maximum body weight of 46.9 g in DDDAM mice was greater than that of 39.7 g in DDDAF mice. In contrast, the maximum body weight of females was greater than that of males of the same strain in *A*^*y*^ mice. The maximum body weight of 72.3 g in DDDYF mice was greater than that of 43.4 g in DDDYM mice. The greatest maximum body weight of non-*A*^*y*^ mice was lesser than the smallest maximum body weight of *A*^*y*^ mice, which indicated that the *A*^*y*^ allele had a strong effect on growth irrespective of strain or sex.

Because the maximum body weight of 50.9 g in B6YM mice was greater than that of 33.3 g in B6AM mice, the maximum body weight of DDDYM mice should have been greater than that of DDDAM mice. However, the maximum body weight of DDDYM mice was lesser than that of DDDAM mice. In model 3, the growth of DDDYM mice stopped between 16 and 17 weeks, following which their body weight declined. In contrast, the body weight of DDDYF mice continued to increase during the observation period (Figure 2B).

Based on these results, we concluded that the growth of DDDYM mice was quite peculiar among the 16 categories of mice used in this study. This peculiar growth was supported by the following evidence: (1) among the 16 categories of mice, the growth rate was the greatest using model 1; (2) the adjusted R-squared value was the smallest using model 2; and (3) the quadratic term [Log(Week)]^2^ was the smallest using model 3. Most importantly, DDDYM mice attained their maximum body weight at 16.7 weeks of age, following which their body weight declined. Mice in the other 15 categories did not attain their maximum body weights during the observation period. Therefore, the lower body weight of DDDYM mice was a consequence of body weight loss.

### Diabetes mellitus is a possible cause of body weight loss in DDD-*A*^*y*^ males

Diabetes and obesity are common characteristics of *A*^*y*^ mice. Hyperglycemia/type 2 diabetes is a possible reason for body weight loss in these mice. Therefore, BGLC levels were determined to address this possibility. DDD-*A*^*y*^ males showed marked hyperglycemia (Figure 3A). DB F_1_-*A*^*y*^ males also showed hyperglycemia but did not lose their body weight, suggesting that hyperglycemia was not the sole cause of body weight loss. Because body weight loss and hyperglycemia were not observed in BD F_1_-*A*^*y*^ males, loci homozygous for DDD alleles must have caused the body weight loss and the maternally derived DDD genome must have induced hyperglycemia.

We also determined plasma lipid levels because dyslipidemia is frequently associated with diabetes and obesity. Clearly, DDD-*A*^*y*^ and DDD mice had higher plasma TG levels than B6-*A*^*y*^ and B6 mice for both the sexes (Figure 3B). In particular, DDD-*A*^*y*^ males had significantly higher plasma TG levels than the other strains, except for DB F_1_-*A*^*y*^ males. In contrast, DDD-*A*^*y*^ and DDD mice were not more cholesterolemic compared with B6-*A*^*y*^ and B6 mice for both the sexes (Figure 3C). Conversely, DDD-*A*^*y*^ males had significantly lower plasma CHO levels than DDD and B6-*A*^*y*^ males.

We determined HMW adiponectin levels in parental strains. Adiponectin is an adipokine secreted from adipose tissues and is known to reverse insulin resistance associated with obesity by stimulating glucose utilization and fatty-acid oxidation [21,22]. Disrupting adiponectin production and/or release causes insulin resistance and increases serum TG levels but not serum CHO levels [23]. Blood HMW adiponectin levels more definitively reflect BMI, the effect of body weight loss, glucose tolerance, insulin sensitivity in the liver, metabolic syndrome, and type 2 diabetes mellitus than the levels of total adiponectin. Clearly, HMW adiponectin levels were significantly reduced in DDD-*A*^*y*^ males.

We also performed histological examinations for possible abnormalities associated with body weight loss. Liver lesions are possible associated with the advancement of obesity. Unexpectedly, liver lesions were more severe in B6-*A*^*y*^ mice than in DDD-*A*^*y*^ mice. In addition, in our earlier observation, we detected enlarged kidneys as a consequence of dilatation of the renal pelvis in DDD-*A*^*y*^ males; however, we failed to confirm this in a subsequent study. Dilatation of the renal pelvis immediately suggested that the enlarged kidney could be caused by hydronephrosis. Nakajima et al. [24] reported a high incidence of hydronephrosis in DDD males, although they did not report any evidence of body weight loss. According to Goto et al. [25], hydronephrosis in DDD mice is heritable and probably controlled by polygenes. Although our DDD strain was a descendant of the DDD strain used by Nakajima et al. [24] and Goto et al. [25], we did not observe hydronephrosis in our DDD strain. Overall, we could not find any histological evidence that may be associated with body weight loss in DDD-*A*^*y*^ males.

Based on these observations, we concluded that DDD-*A*^*y*^ males suffered from diabetes mellitus.

### Body weight loss is heritable and controlled by a major QTL on chromosome 4

When the agouti locus genotype was included as a covariate, a significant QTL × covariate interaction was identified on chromosome 4. The importance of this QTL was that it was significant for body weight and BMI and it interacted with the *A*^*y*^ allele. That is, the action of this QTL differed between BC non-*A*^*y*^ and BC *A*^*y*^ males, which suggested a reason why body weight loss occurred in DDD.Cg-*A*^*y*^ males but not in DDD males. This QTL was also important because it was suggestive QTLs for HMW adiponectin levels and BGLC and PGLC levels. The DDD allele was associated with decreased body weight, BMI, and HMW adiponectin levels and increased BGLC and PGLC levels, suggesting that the QTL on chromosome 4 was responsible for diabetes mellitus. For PGLC, a larger QTL effect was identified on chromosome 12. However, this QTL was unlikely to be a direct cause of hyperglycemia of DDD.Cg-*A*^*y*^ males because the B6 allele was associated with increased glucose level at this locus. It was uncertain whether this locus was allelic with that for TG levels because the B6 allele was associated with decreased TG levels.

The QTL on chromosome 1 contained *Ifi202b* and the QTL on chromosome 4 contained *Zfp69* within the 95% CI as a strong candidate gene [19,20]. *Ifi202b* was identified as a cause of *Nob3*, a major QTL for obesity and hyperglycemia in an F_2_ intercross between NZO and B6 mouse strains [26]. In B6 mice, *Ifi202b* is disrupted by a deletion of its 5’-flanking region including exon 1B and the promoter [19]. According to the PCR analysis, the DDD strain had exon 1B as opposed to B6 allele. There is no reason to exclude *Ifi202b* as a candidate gene for the QTL on chromosome 1; however, we are still uncertain whether the QTL on chromosome 1 is allelic to *Nob3* because of following reasons. First, the phenotypic effect of the QTL on chromosome 1 on body weight, BGLC, and PGLC was apparently weak as compared with *Nob3*. Second, the B6 allele was associated with increased body weight at the present QTL, whereas the B6 allele was associated with decreased body weight in (NZO × B6) F_2_ population.

On the other hand, *Zfp69* was identified as a cause of *Nidd/SJL* in a backcross male progeny between NZO and SJL strains [27]. The *Zfp69* gene is disrupted in B6 and NZO strains by an insertion of a retrotransposon sequence [20,28]. Intriguingly, disrupted allele protects mice against diabetes. Thus, in a cross between NZO and SJL mice, the wild-type SJL allele contributed to diabetes. The SJL allele at *Nidd/SJL* was associated with decreased body weight and BMI and increased BGLC and TG levels. In the similar way as did the SJL allele at *Nidd/SJL*, the DDD allele at the QTL on chromosome 4 was associated with decreased body weight and BMI and increased BGLC, suggesting the allelism between the two QTLs, However, our results of *Zfp69* genotyping suggested that the exon 3A was present in the DDD strain, and that there were no differences between the B6 and DDD alleles. In addition, we sequenced all exons except for exon 3A in the DDD strain and found there were no differences between the B6 and DDD alleles (data not shown). Different from the effects associated with *Nidd/SJL*, the QTL on chromosome 4 in our cross did not have any substantial effects on TG levels. Thus, the QTL on chromosome 4 was unlikely to be caused by the *Zfp69*. However, this by no means rule out the possibility that the *Zfp69* plays a role in parental DDD.Cg-*A*^*y*^ males because the diabetogenic effect of the *Zfp69* variant is markedly dependent on interaction with other genes of the background strain [20].

With regard to the QTL on chromosome 4, we can postulate two other candidate genes that are closely related to body weight and diabetes mellitus. One is coristatin (*Cort*), which is located at 149.13 Mbp [29]. Although the body weight and blood glucose levels did not significantly differ between *Cort* deficient (*Cort*^*−/−*^) and wild type (*Cort*^*+/+*^) mice, *Cort*^*−/−*^ males, but not females, showed insulin resistance during insulin tolerance test. The other candidate gene is hexose-6-phosphate dehydrogenase (*H6pd*), which is located at 150 Mbp [30]. Fasting glucose level was significantly lower in *H6pd* deficient (*H6pd*^*−/−*^) mice compared with wild type (*H6pd*^*+/+*^) mice. At 10 weeks of age, *H6pd*^*−/−*^ mice were significantly lighter than *H6pd*^*+/+*^ mice. These are undoubtedly candidate genes for the QTL; however, we are still uncertain whether they are causative of the QTL because the effect of the QTL on chromosome 4 is only apparent in the presence of the *A*^*y*^ allele. That is, we cannot correctly assess their candidacy unless these mutant phenotypes are investigated in the presence of the *A*^*y*^ allele.

### PGLC and HMW adiponectin levels are critical factors that affect the body weight of BC *A*^*y*^ males

Our best-fit linear model included PGLC and HMW adiponectin levels as linear or quadratic terms, which suggested that PGLC and HMW adiponectin levels were critical factors that affected body weights of BC *A*^*y*^ males as opposed to CHO and TG levels. These results were in accordance with those of our QTL analyses.

Based on partition method analysis, nine abnormal BC *A*^*y*^ males exhibited body weight loss similar to DDD-*A*^*y*^ males. Furthermore, the trajectory of the growth curve of abnormal BC *A*^*y*^ males was very similar to that of DDD-*A*^*y*^ males. This suggested that body weight loss in abnormal BC *A*^*y*^ males was caused by the same factors that contributed to body weight loss in DDD-*A*^*y*^ males. When the phenotypes were compared between the two groups of BC *A*^*y*^ males, body weight, BMI, BGLC, PGLC, and HMW adiponectin levels significantly differed between abnormal and normal mice. In contrast, CHO and TG levels did not differ significantly between these two groups. This suggests again that PGLC and HMW adiponectin levels are critical factors that affect the body weight of BC *A*^*y*^ males. Furthermore, the nine abnormal BC *A*^*y*^ males and DDD-*A*^*y*^ males may have shared the same genetic basis for body weight loss because seven of the nine abnormal BC *A*^*y*^ males were homozygous for the DDD allele at *D4Mit234*, a microsatellite marker closest to the QTL on chromosome 4.

### QTL on chromosome 4 as a modifier of the *A*^*y*^ allele

As mentioned at the beginning of the Background section, five single gene obesity mutations are known at present. These mutations cause obesity and diabetes in mice. The phenotypic effects of these mutations are very strong; nevertheless, the phenotypes caused by these mutations are substantially influenced by the genetic backgrounds on which these mutations are placed [31-36]. In particular, Coleman et al. [31-33] studied the effects of the *Lep*^*ob*^ and *Lepr*^*db*^ mutations on B6 and C57BL/KsJ (KSJ) backgrounds and developed the concept of the diabetes-sensitive and the diabetes-resistant backgrounds. B6 is the resistant background to diabetes associated with obesity caused by the *Lep*^*ob*^ and *Lepr*^*db*^ mutations, whereas KSJ is the diabetes-sensitive background. Similarly, if we assume that DDD is the sensitive background, B6 is again defined as the resistant background to diabetes associated with obesity caused by the *A*^*y*^ allele. Therefore, the QTLs on chromosomes 1 and 4 are expected to encode genes that constitute the diabetes-sensitive genetic background.

## Conclusions

Lower body weight of DDD.Cg-*A*^*y*^ males was confirmed to be a consequence of body weight loss. Although we did not yet establish convincing evidence, diabetes mellitus was suggested to be a possible contributing factor that caused body weight loss. Body weight loss was a heritable phenomenon, and a QTL on distal chromosome 4 was suggested to be a major factor responsible for this. This QTL interacts with the *A*^*y*^ allele, suggesting a reason why body weight loss occurs in DDD.Cg-*A*^*y*^ males but not in DDD males.

## Competing interest

The authors declare that they have no competing interests.

## Authors’ contributions

JS and KS conceived the study. JS conducted the experiments on mice. KS conducted statistical analysis for growth. JS conducted QTL mapping analysis. JS and KS wrote the manuscript. Both authors read and approved the final manuscript.
